# Supplementary material for: Identifying SARS-CoV-2 antiviral compounds by screening for small molecule inhibitors of nsp13 helicase
Source: Biochem J. 2021 Jul 2;478(13):2405–23. doi: 10.1042/BCJ20210201 (PMC8286831; doi:10.1042/BCJ20210201)
Supplement: Supplementary Figures S1-S11 [file BCJ-478-2405-s1.pdf]

Supplementary Figure legends

Supplementary Figure S1. SARS-CoV-2 nsp13 protein expression and FRET-based helicase assay development and optimization. (**A**, **B**) FH-nsp13 and GST-nsp13 were purified by gel filtration on a Superdex 200 Increase 10/300 GL column (S200I) and fractions were analysed by SDS-PAGE and Coomassie staining. (**C**) Emission of fluorescence by 50 nM Cy3 strand could be efficiently quenched by 55 nM BHQ-2 strand after the annealing protocol. (**D**) Annealing dynamics of the Cy3 strand and BHQ strand at room temperature. Without the competitor strand (0 competitor), 50 nM Cy3 strand slowly annealed with 50 nM BHQ-2 strand resulting in quenching of fluorescence by approximately 40% over 30 min. Adding increasing fold of the competitor strand (relative to 50 nM) results in less quenching by disrupting spontaneous annealing between the Cy3 and BHQ strands. (**E**, **F**) Unwinding of 50 nM DNA duplex by 1 nM FH-nsp13 was observed with a duplex with 5' dT overhang but not with a duplex with 3' dT overhang. (**G**) A native gel-based assay was used to visualise unwinding of DNA duplexes by nsp13. The 5' end of the longer strand of the DNA duplex was radiolabelled. Strand separation of the duplex by nsp13 was observed over time.

Supplementary Figure S2. SARS-CoV-2 nsp13 enzyme kinetics. (**A-B,D-E**) Nsp13 activity was tested with increasing concentrations of ATP or the nucleic acid substrates to determine the Michaelis constants (K_m) shown in Figure 2. (**A**) DNA substrate titration. Conditions: 1 nM FH-nsp13, 1 mM ATP, indicated DNA substrate concentrations. (**B**) RNA substrate titration. Conditions: 1 nM FH-nsp13, 1 mM ATP, indicated RNA substrate concentrations. (**C**) A native gel-based assay was used to visualise unwinding of RNA duplexes by FH-nsp13. The 5' end of the longer strand of the RNA duplex was radiolabelled. Strand separation of the duplex by nsp13 was observed over time. (**D**) ATP titration in the presence of a DNA substrate. Conditions: 2 nM FH-nsp13, 200 nM DNA substrate, indicated ATP concentrations. (**E**) ATP titration in the presence of the RNA substrate. Conditions: 3 nM FH-nsp13, 200 nM RNA substrate, indicated ATP concentrations. (**F**) Addition of up to 5% DMSO did not disrupt nsp13 activity or annealing properties of nucleic acid substrates.

Supplementary Figure S3. High-throughput SARS-CoV-2 nsp13 inhibitor screen. (**A**) Layout of 384-well plates used in the HTS. (**B**) Representative time-course data showing nsp13 DNA duplex unwinding activity in a 384-well plate format as used in the HTS. (**C, D**) Normalised initial velocity values for all the compounds in the HTS, before positional correction. (**E, F**) Normalised endpoint signals for all the compounds in the HTS. (**G**) Distribution of Z-scores of all the compounds tested in the 6.25 μM screen.

Supplementary Figure S4. Concentration-response curves for validation of selected hits identified in the SARS-CoV-2 nsp13 HTS. The experiment (blue) was performed with 180 nM fluorogenic DNA substrate, 0.1 mM ATP, 1.5 nM nsp13 and in the absence of detergent. Fluorescence quenching properties of the compounds were tested at the same compound concentrations to rule out false positives that interfered with fluorescence emission (grey).

Fluorescence intensities were measured using 180 nM unannealed Cy3 strand in the absence of a quencher strand.

Supplementary Figure S5. Concentration-response curves for validation of selected hits identified in SARS-CoV-2 nsp13 HTS. The experiment (blue) was performed using 180 nM fluorogenic RNA substrate, 2 mM ATP, 1 nM nsp13 and in the presence of 0.02% Tween-20. Fluorescence quenching properties of the compounds were also tested (grey) to rule out false positives that interfered with fluorescence emission.

Supplementary Figure S6. Concentration-response curves for validation of selected hits identified in SARS-CoV-2 nsp13 HTS. The experiment was performed using 180 nM fluorogenic DNA substrate, 0.1 mM ATP, 0.5 nM nsp13 in the presence (red) or absence (blue) of 0.02% Tween-20 in the reaction.

Supplementary Figure S7. Concentration-response curves of SARS-CoV-2 nsp13 and RdRp activity for selected compounds. Nsp13 helicase activity was assessed using the helicase assay described in this manuscript and was performed with 100 nM fluorogenic RNA substrate, 0.3 mM ATP, 3 nM nsp13 in RdRp assay buffer (see below) with 0.02% Tween-20. RdRp activity was measured using the strand displacement (SD) assay described in detail in Bertolin et al (Biochem J, this issue) and was performed with 100 nM of fluorogenic RNA substrate, 100 nM of RdRp complex (Sf nsp12-3F/7L8 preincubated with Sf 7H8 at a 1:3 ratio) in RdRp assay buffer (20 mM HEPES pH 7.5, 10 mM KCl, 1 mM DTT, 5 mM $MgCl_2$, 2 mM $MnCl_2$, 0.1 mg/ml BSA) with 0.02% Tween-20. Nsp13 activity in the helicase assay is depicted in red and RdRp activity in the SD assay is depicted in green.

Supplementary Figure S8. Concentration-response curves for validation of selected hits identified in SARS-CoV-2 nsp13 HTS. (**A**) The experiment was performed with 180 nM fluorogenic RNA substrate, 0.1 mM ATP, 1 nM nsp13 in the presence (red) or absence (blue) of 0.02% Tween-20. (**B**) Fluorescence quenching properties of the compounds were also tested (grey) to rule out false positives that interfered with fluorescence emission.

Supplementary Figure S9. Concentration-response curves for validation of selected hits identified in SARS-CoV-2 nsp13 HTS. The experiment was performed using 180 nM fluorogenic RNA substrate, 0.1 mM ATP, 1 nM nsp13 in the presence (red) or absence (blue) of 0.01% Triton X-100.

Supplementary Figure S10. Chemical structures of selected nsp13 inhibitors. FPA-124 data is shown in Table 2. Suramin, NF 023, PPNDS, Evans Blue, Diphenyl Blue, PDK1/Akt/Flt Dual Pathway Inhibitor data are shown in Table 1.

Supplementary Figure S11. Cytotoxicity analysis and comparative dose–response curves of selected antiviral compounds against SARS-CoV-2 in cell culture. (**A**) SSYA10-001, myricetin, suramin and FPA-124 cytotoxicity analysis of DRAQ7-stained Vero E6 cell. Representative images of one experiment in triplicate shown. (**B**) Anti-SARS-CoV-2 activities of SSYA10-001, myricetin, suramin and FPA-124 in combination with 1 µM remdesivir. Left, representative images showing N protein immunofluorescence (green) and DRAQ7 staining (red) of one experiment in triplicate shown. Right, dose-response curve analysis. Viral infection was calculated as the area of viral plaques stained for N protein and cell viability as the area of cells stained for DRAQ7. Data is plotted as percentage compared to 1 µM remdesivir only treated wells (100%). Values represent mean and standard deviation (SD) of three replicates. FIJI software was used to calculate areas and Prism software to calculate EC_{50} values.

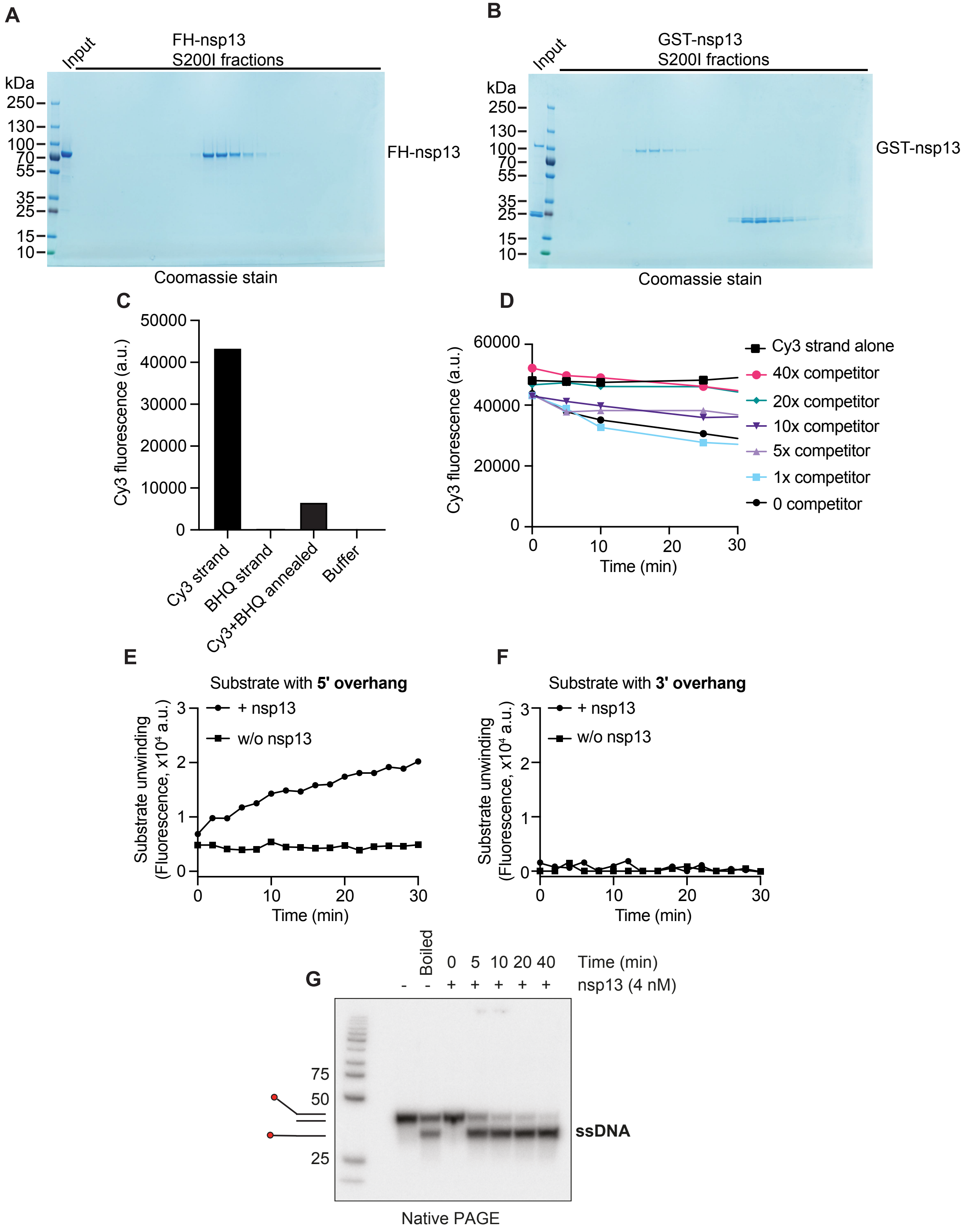

nsp13 manuscript - Supplementary Figure S1

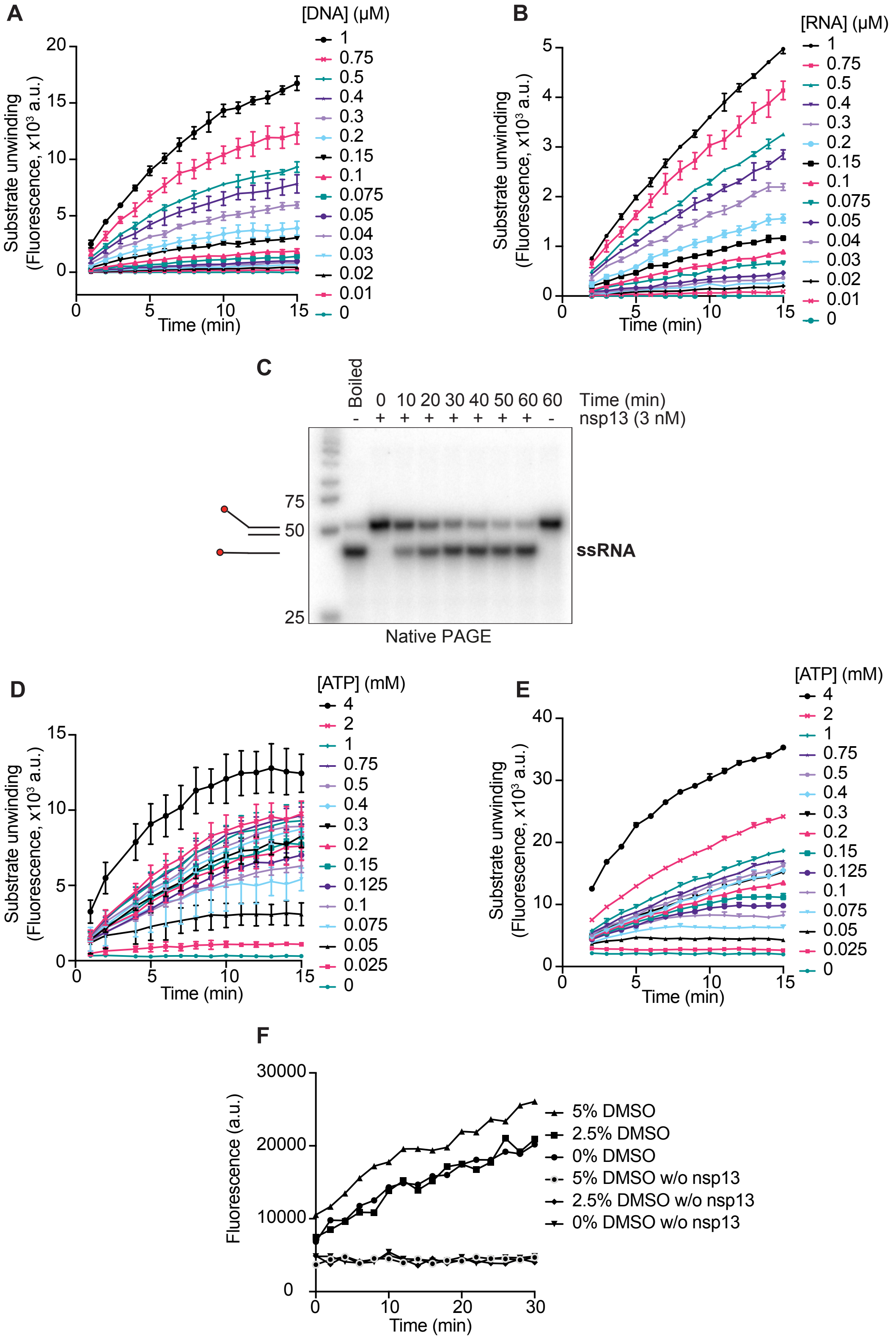

nsp13 manuscript - Supplementary Figure S2

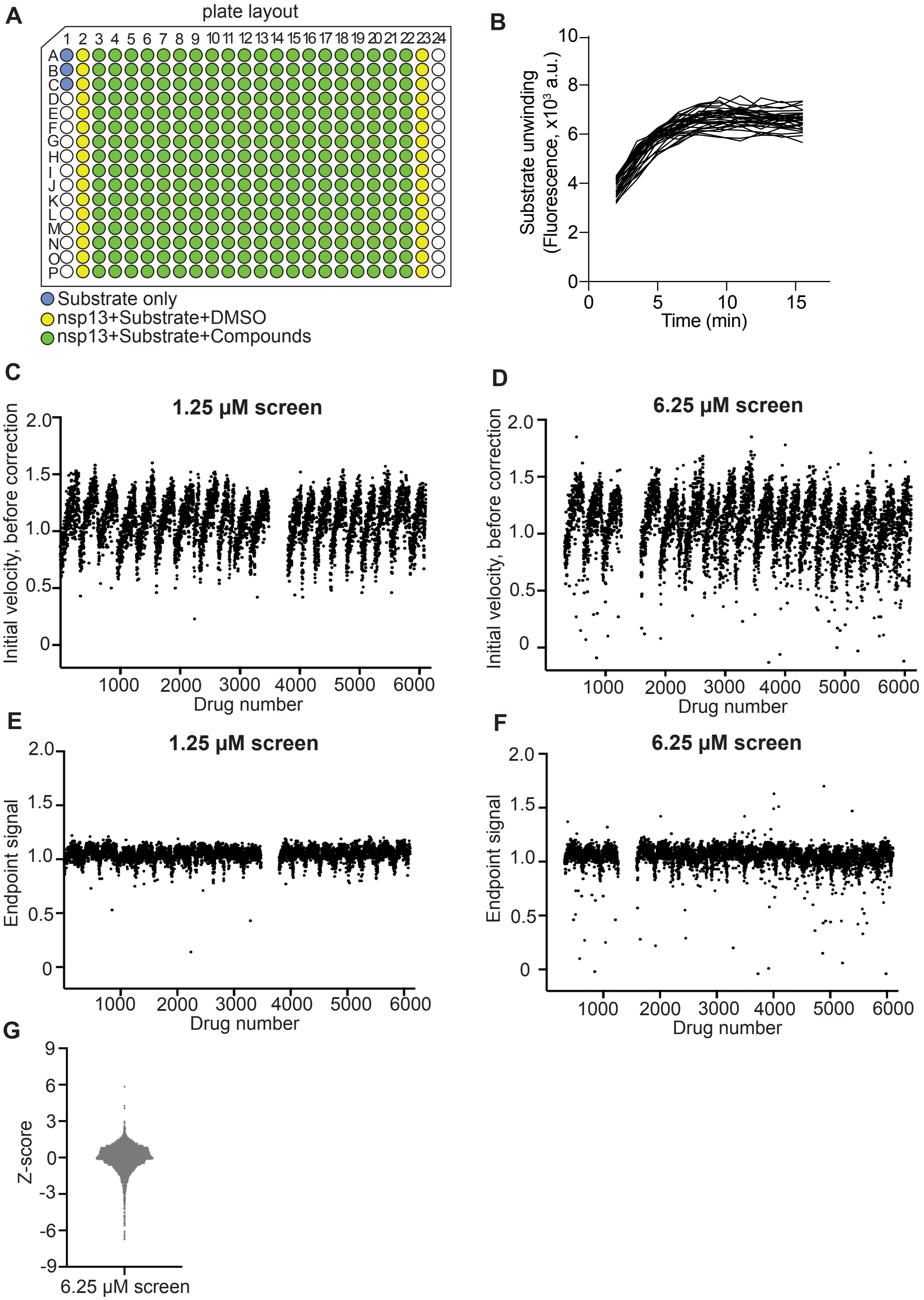

nsp13 manuscript - Supplementary Figure S3

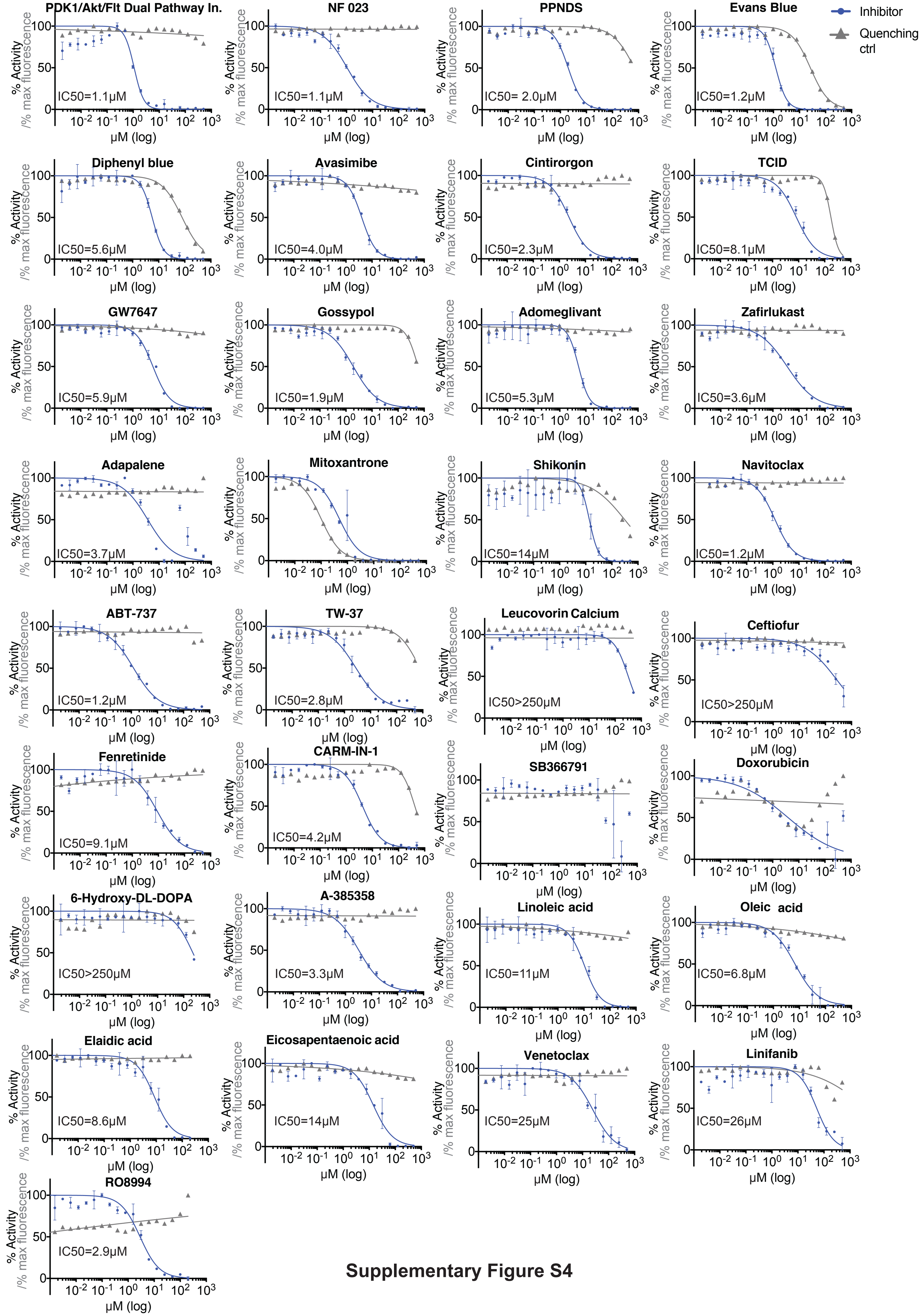

Supplementary Figure S4

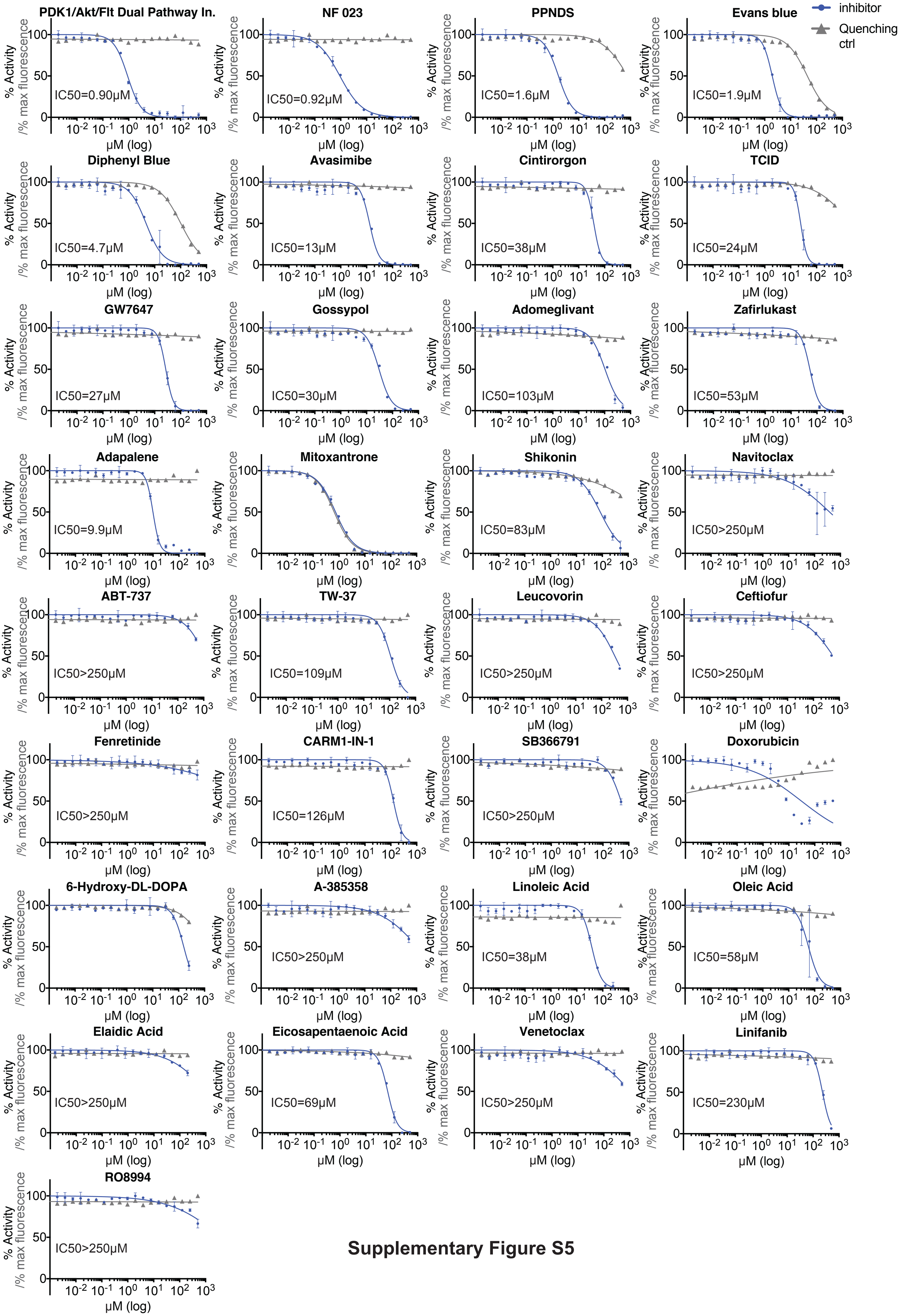

Supplementary Figure S5

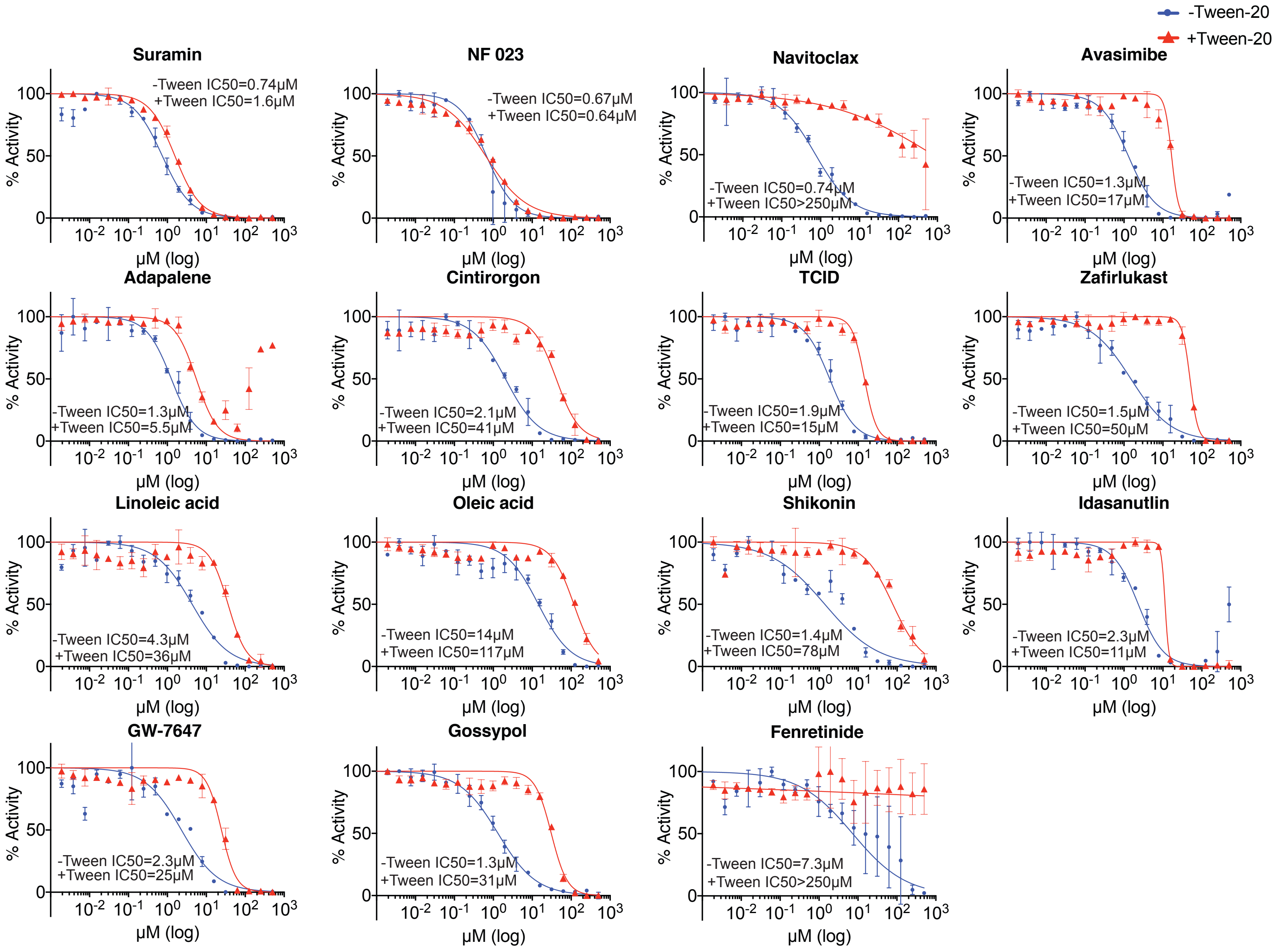

Supplementary Figure S6

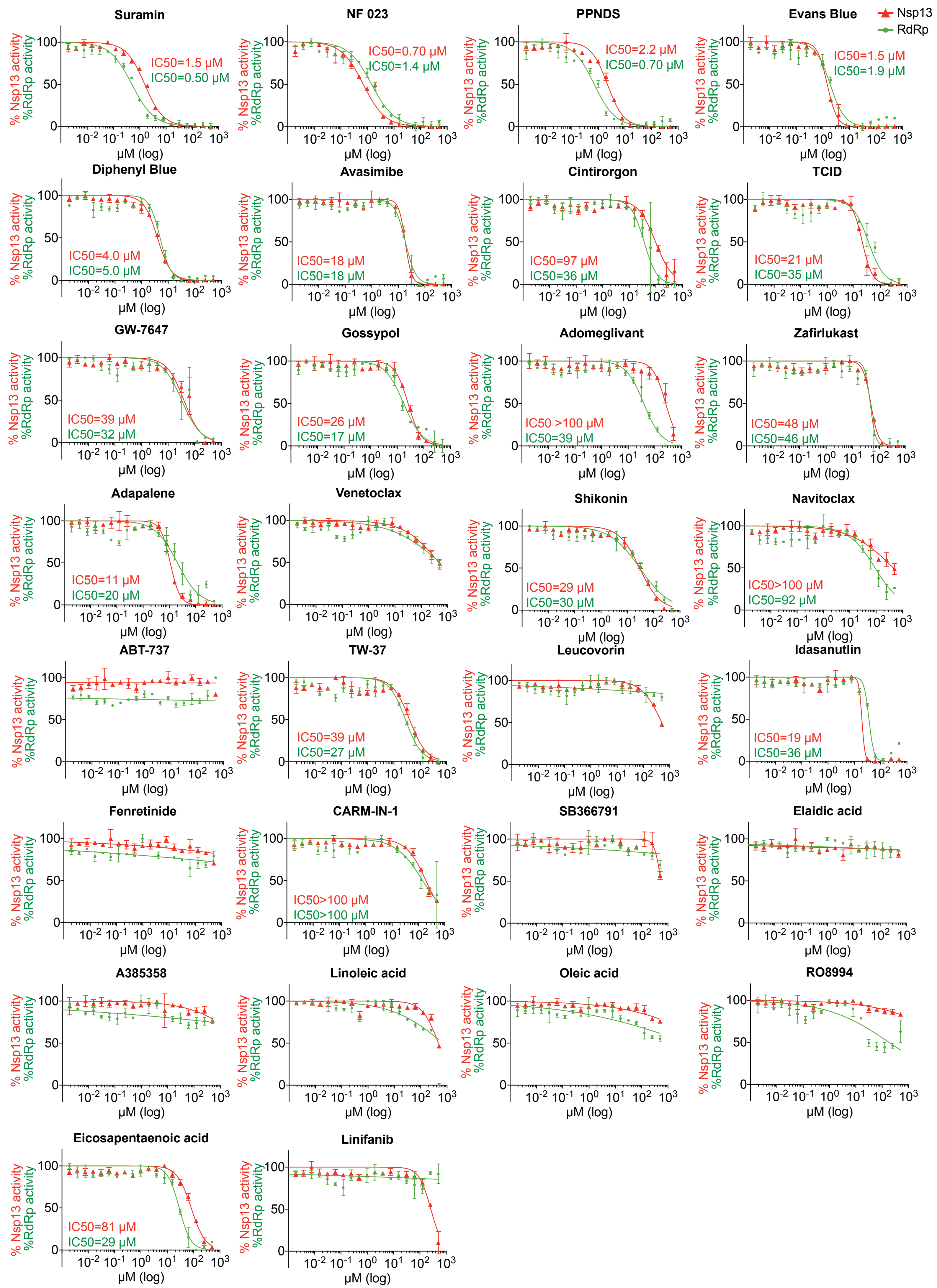

Supplementary Figure S7

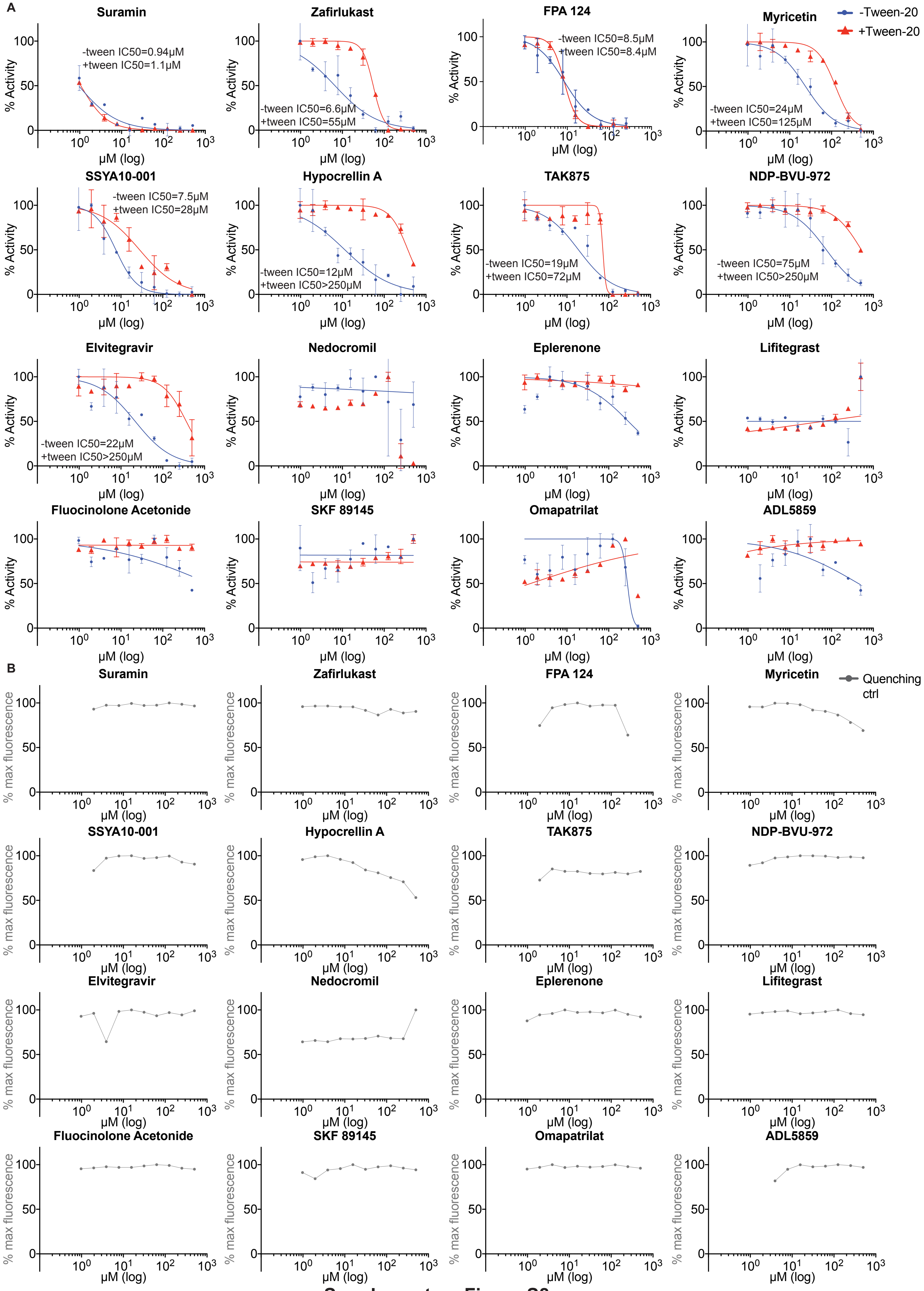

Supplementary Figure S8

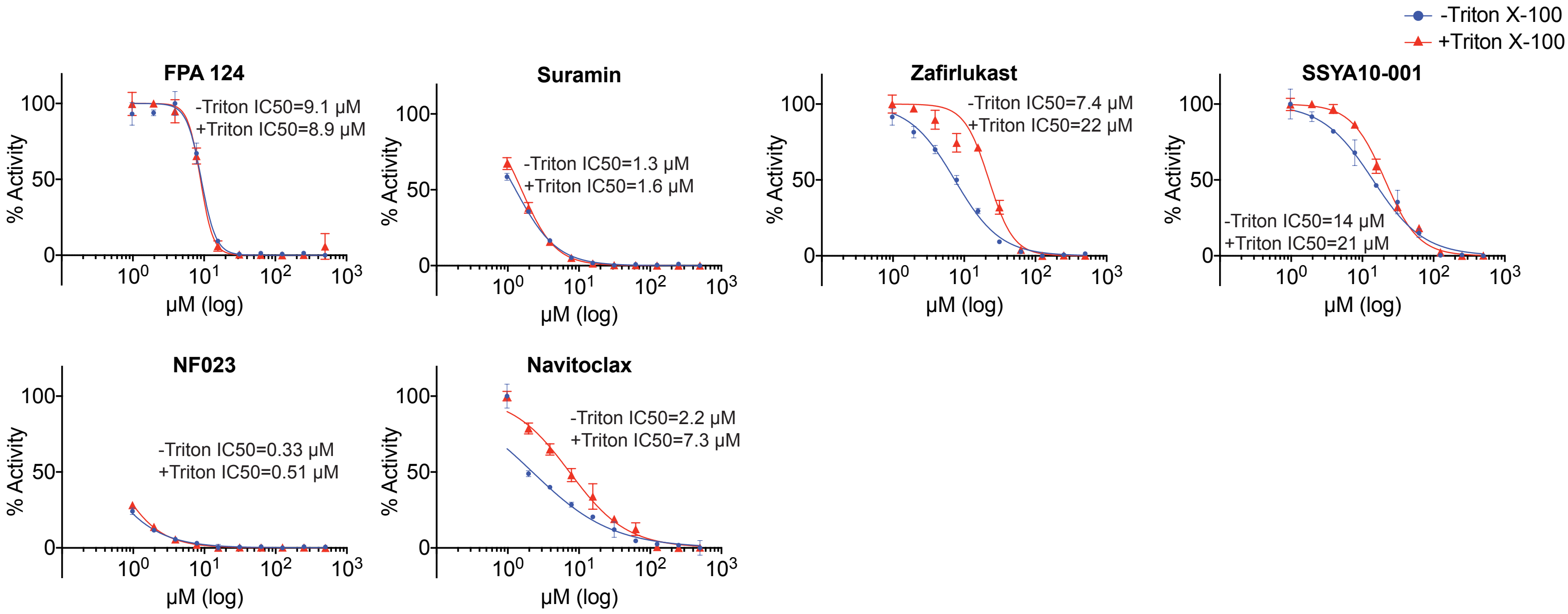

Supplementary Figure S9

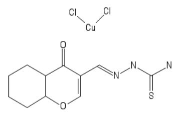

FPA-124

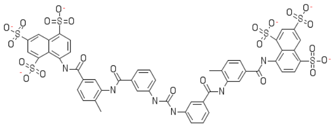

Suramin

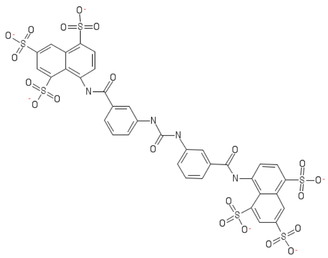

NF 023

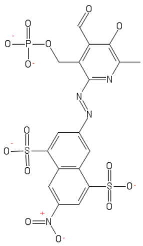

PPNDS

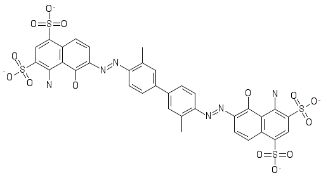

Evans Blue

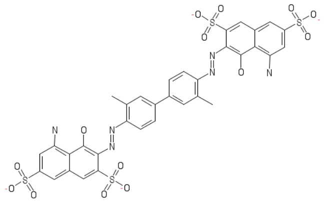

Diphenyl Blue

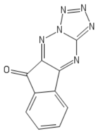

PDK1/Akt/Flt Dual Pathway Inhibitor

nsp13 manuscript - Supplementary Figure S10

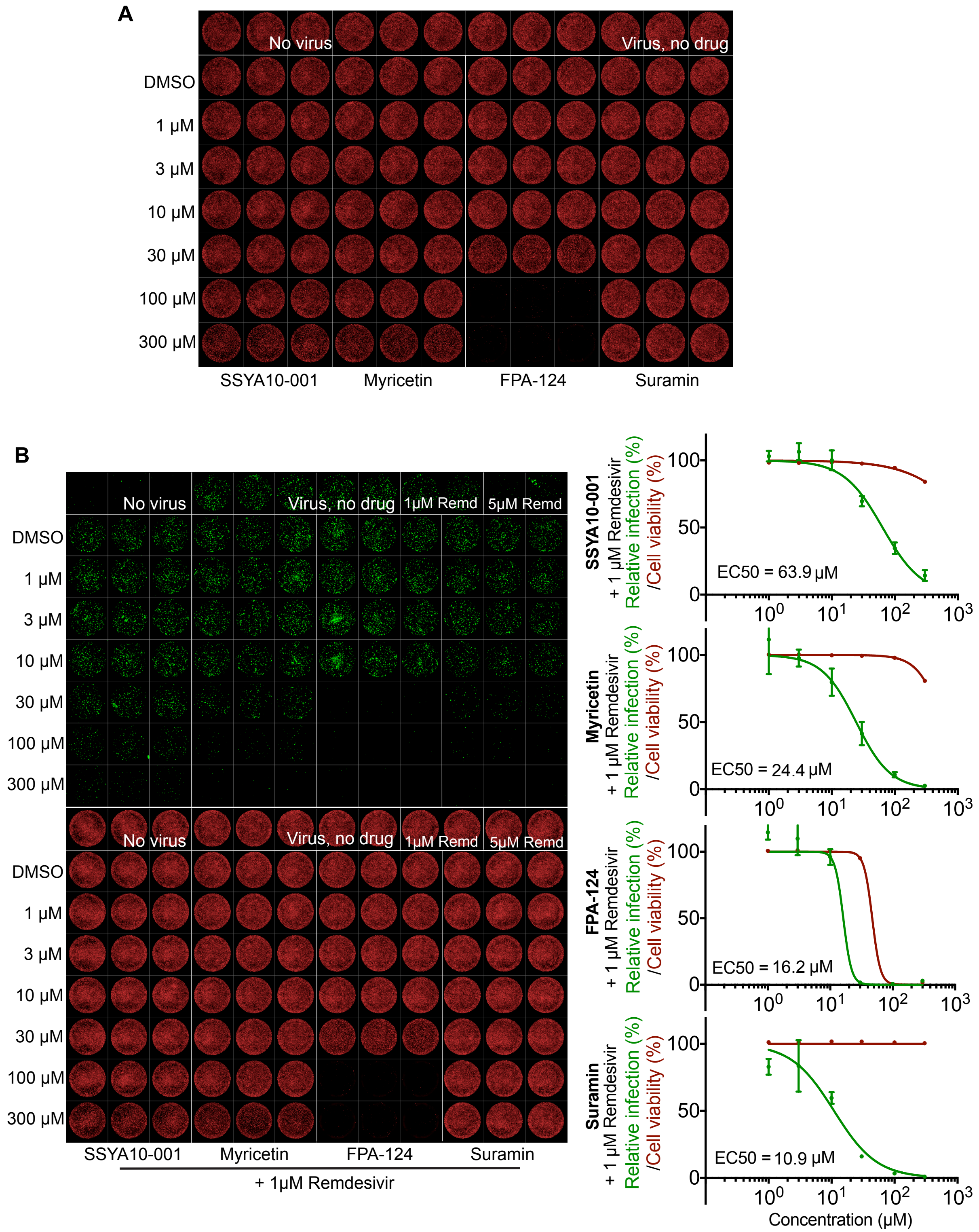

nsp13 manuscript - Supplementary Figure S11
